# German health interview and examination survey for adults (DEGS) - design, objectives and implementation of the first data collection wave

**DOI:** 10.1186/1471-2458-12-730

**Published:** 2012-09-01

**Authors:** Christa Scheidt-Nave, Panagiotis Kamtsiuris, Antje Gößwald, Heike Hölling, Michael Lange, Markus A Busch, Stefan Dahm, Rüdiger Dölle, Ute Ellert, Judith Fuchs, Ulfert Hapke, Christin Heidemann, Hildtraud Knopf, Detlef Laussmann, Gert BM Mensink, Hannelore Neuhauser, Almut Richter, Anke-Christine Sass, Angelika Schaffrath Rosario, Heribert Stolzenberg, Michael Thamm, Bärbel-Maria Kurth

**Affiliations:** 1 Department of Epidemiology and Health Monitoring, Robert Koch Institute, General-Pape-Strasse 62-66, 12101 Berlin, Germany

**Keywords:** Health, Indicators, Survey, Examination, Adults, Germany, Population, Trends, Follow-up

## Abstract

**Background:**

The German Health Interview and Examination Survey for Adults (DEGS) is part of the recently established national health monitoring conducted by the Robert Koch Institute. DEGS combines a nationally representative periodic health survey and a longitudinal study based on follow-up of survey participants. Funding is provided by the German Ministry of Health and supplemented for specific research topics from other sources.

**Methods/design:**

The first DEGS wave of data collection (DEGS1) extended from November 2008 to December 2011. Overall, 8152 men and women participated. Of these, 3959 persons already participated in the German National Health Interview and Examination Survey 1998 (GNHIES98) at which time they were 18–79 years of age. Another 4193 persons 18–79 years of age were recruited for DEGS1 in 2008–2011 based on two-stage stratified random sampling from local population registries. Health data and context variables were collected using standardized computer assisted personal interviews, self-administered questionnaires, and standardized measurements and tests. In order to keep survey results representative for the population aged 18–79 years, results will be weighted by survey-specific weighting factors considering sampling and drop-out probabilities as well as deviations between the design-weighted net sample and German population statistics 2010.

**Discussion:**

DEGS aims to establish a nationally representative data base on health of adults in Germany. This health data platform will be used for continuous health reporting and health care research. The results will help to support health policy planning and evaluation. Repeated cross-sectional surveys will permit analyses of time trends in morbidity, functional capacity levels, disability, and health risks and resources. Follow-up of study participants will provide the opportunity to study trajectories of health and disability. A special focus lies on chronic diseases including asthma, allergies, cardiovascular conditions, diabetes mellitus, and musculoskeletal diseases. Other core topics include vaccine-preventable diseases and immunization status, nutritional deficiencies, health in older age, and the association between health-related behavior and mental health.

## Background

In Germany, several health interview and examination surveys have been carried out since 1984. These surveys addressed different subsets of the population and were conducted at irregular intervals. In the framework of the German Cardiovascular Prevention Study (GCP), residents of former West Germany 25–69 years of age were surveyed in three independent consecutive surveys conducted in 1984–86 (n = 4790), 1987–89 (n = 5335), and 1990–91 (n = 5311). The surveys were restricted to the population in private households and to those who could sufficiently speak German [1]. In 1991–92, the Survey East was conducted, in order to provide comparable health data for adults 18–79 years residing in private households of former East Germany. Data of the Survey East and the 1990–91 GCP survey were pooled for analyses of health data in the reunified Germany. Nearly a decade later, the German National Health Interview and Examination Survey 1998 (GNHIES98) conducted under the auspices of the Robert Koch Institute (RKI) was the first nationally representative health survey of non-institutionalized adults 18–79 years in reunified Germany [2]. It opened the perspective for analyzing regional differences in health trends in comparison to the 1990–92 data [3]. In 2003–2006, the RKI conducted the German Health Interview and Examination Survey for Children and Adolescents (KiGGS). Including a total of 17641 study participants, KiGGS established a large nationally representative health database for the non-institutionalized German population 0–17 years of age [4].

In 2008, the German Ministry of Health commissioned the RKI to implement a system of health studies for continuous health monitoring of the non-institutionalized population [5]. Survey components include: (1) annual health interview surveys (German Health Update, GEDA) conducted in large representative and independently drawn samples of the population 18 years and older, (2) a national cohort study of children and adolescents in Germany based on continuous follow-up of the KiGGS study population, (3) periodically repeated interview and health examination surveys of the population 18–79 years of age (German Health Interview and Examination Survey for Adults, DEGS). We here describe the design, objectives and implementation of the first wave of data collection in DEGS (DEGS1), which was carried out from November 2008 to November 2011.

## Methods/design

### Study design

DEGS is primarily designed as a periodically repeated national health interview and examination survey of adults in Germany. The target population comprises adults 18–79 years of age with permanent residence in Germany according to local population registries. In order to lay the ground for longitudinal studies, persons who had participated in the 1998 national health interview and examination survey (GNHIES98) were invited to take part in DEGS1, provided they had agreed to be recontacted and were still contactable [6]. Representativeness of DEGS1 as a cross-sectional survey is maintained by extending the population sample based on a two-stage sampling procedure according to power analyses and sample size estimations as described below.

The implementation of DEGS1 conforms to the principles outlined in the Declaration of Helsinki [7] and to the German Federal Data Protection Act. The DEGS1 study protocol was consented with the Federal and State Commissioners for Data Protection and approved by the Charité-Universitätsmedizin Berlin ethics committee in September 2008 (No. EA2/047/08). Participants provided written informed consent prior to the interview and examination.

### Study population

Overall 17410 persons, of whom N = 6402 or 37% were GNHIES98 participants, were invited to participate in DEGS1. After excluding non-deliverable survey contacts, persons unable to provide written informed consent and persons with insufficient German language skills, the overall net sample consisted of 16305 adults, of whom N = 6358 or 39% were GNHIES98 participants. Overall, 8152 adults (4283 women; 3869 men) participated in DEGS1. Total numbers of the study population and response rates are summarized by age group and GNHIES98 participation status in Table 1. Response rates were consistently higher among GNHIES98 participants compared to newly sampled individuals.

**Table 1:** Study population and response rates in DEGS1

	**Total**	**GNHIES98 participants**		**Newly sampled participants**	
**Age group (yrs)**	**N**	**N**	**Response**	**N**	**Response**
18–29	1073	19^a^		1054	40%
30–39	1014	427	49%	587	40%
40–49	1539	901	63%	638	44%
50–59	1592	928	69%	664	46%
60–69	1537	867	73%	670	47%
70–79	1233	653	65%	580	38%
**18–79**	**7988**	**3795**	**64%**	**4193**	**42%**
80–91	164	164	38%		
**18–91**^**a**^	**8152**	**3959**	**62%**	**4193**	**42%**

^a^GNHIES98 participants were 28–91 years of age at the time of DEGS1.

Both interview and examination data are available for 89% of the study population (N = 7238; 3765 women, 3473 men). The remainder of the study population (N = 914; 518 women, 396 men) have interview data only, because they were unable or unwilling to come to one of the local study centers. Cross-sectional and trend analyses will be restricted to 7988 DEGS1 participants 18 to 79 years of age, with examination as well as interview data available for 7116 of these. Longitudinal studies will include a total of 3959 persons 28–91 years of age who participated in DEGS1 as well as in the GNHIES98.

### Sampling procedure

The DEGS1 sampling protocol was developed in co-operation with the Leibniz Institute for the Social Sciences (GESIS), Mannheim, Germany [8]. Like previous German health interview and examination surveys [1,2,4] DEGS1 is based on two-stage stratified cluster sampling. Primary sampling units (PSUs) are the communities. PSUs were sampled from a list of German communities stratified according to districts and the BIK classification system, which takes into account the grade of urbanization, regional population density, and administrative borders [9]. Sampling was done with probability proportional to community size using the Cox procedure for controlled rounding [10]. Selected metropolitan areas were generally represented by several PSUs. Selected communities with less than 1000 inhabitants were combined with neighboring small communities to form a single PSU. Within PSUs, random samples of individuals, stratified by 10-year age groups, were drawn from local population registers.

Since DEGS1 included GNHIES98 participants, the DEGS1 PSU sample had to build upon a total of 120 PSUs previously sampled for the 1998 survey. In order to remain representative at the population level, additional PSUs (N = 60) were sampled. In newly added PSUs, all participants were newly sampled. Within GNHIES98 PSUs, only newly recruited individuals are included in the age group 18–28 years due to the aging of the GNHIES98 cohort by 10–14 years. Persons 30 years and older were sampled as necessary, in order to replace the number of participants expected to decline participation in DEGS1 or to be lost to follow-up. The number of newly sampled PSUs and individuals per PSU was determined by statistical power and sample size considerations as described below.

### Statistical power and sample size calculations

Given the cluster design, simple variance estimates of prevalence estimates will underestimate total variability and hence estimation error, because individuals within PSUs can be assumed to show greater similarities than individuals randomly selected from the entire population of Germany. The increase in estimation error is quantified by the design effect (*Deff*). Total sample size needs to be increased in proportion to *Deff* to attain the same level of precision as in simple random sampling [11]. *Deff* depends on the average number of individuals within PSUs (*m*) and the degree of similarity within clusters which is estimated by the intra class correlation coefficient (*ICC*): *Deff* = 1 + *ICC*(*m*-1) [12]. It is therefore more efficient to increase the total number of PSUs rather than *m,* in order to keep the total sample size as small as possible. Reasonably precise estimation of a sex and 10-year-age group–specific prevalence of 1% (95% CI to be attained: 0.5–2.1%) would require a total sample size of about 7500. In this calculation, *Deff* is ignored as the number of persons in a PSU within any sex-age-group can be expected to be very small. Analyses of GNHIES98 data showed that *ICC* can be assumed to take on values between 0.005 and 0.036. Based on the experience that survey logistics permit the examination of 40–50 persons per PSU, design effects would be 4–15% higher with 50 instead of 40 persons seen per PSU. The required precision could most efficiently be reached based on a total number of 180 PSUs and an average of *m* = 42 persons seen per PSU, yielding a total sample size of 180 × 42 = 7560 [8]. This sample size also proved to provide 90% power to detect a 3% change in overall obesity prevalence in analyses for time trends at the 5% significance level based on two-sided tests [13].

### Recruitment of the study population

Eligible persons were invited to participate in the survey by letter. An information booklet illustrated the study objectives, procedures and logistics. A response card was enclosed to fill in telephone numbers and preferred contact times. Participants were offered a small monetary incentive.

Invitation letters were sent approximately five weeks prior to the survey visit. In order to optimize response rates, survey activities were announced in local newspapers, and if possible, on local television and radio stations. In addition, small survey reports were published at regular intervals in national medical and public health journals. Persons willing to participate were contacted by telephone for scheduling and further information. A confirmation letter including the time of the appointment, location plan, and preparatory instructions was sent thereafter. Persons with appointments in the morning were asked to keep an overnight fast and not eat or drink anything but water before blood testing, unless they were diabetic. Persons with afternoon appointments were asked to fast for at least four hours, unless they were diabetic. Participants were asked to bring along immunization records as well as the original containers of all medications (prescription and over-the-counter) used within 7 days prior to the survey appointment.

Personal contact was sought to persons who had not responded to the invitation letter within four weeks. Exclusion criteria (such as insufficient language proficiency or inability to provide informed consent) were recorded. Persons willing to participate but facing organizational problems (e.g. lack of transportation, time constraints) were offered transportation and flexible scheduling, such as appointments early in the morning or in the evening.

### Field logistics

Fieldwork in DEGS1 extended over three years. Data collection started on November 25, 2008 and ended on November 26, 2011. PSUs were successively visited by two mobile study teams according to a random touring schedule, in order to avoid a systematic bias of study results by seasonal or time trends. Duration of stay at a particular PSU was limited to one week. In order to include a sufficiently high number of study participants per PSU, eligible persons had to be scheduled for appointments ranging from 7:30 a.m. to 6:00 p.m. during the week and from 7:30 a.m. to 10:30 a.m. on Saturdays.

Study teams consisted of specifically trained health professionals, including a study physician and three technicians. Interviews and examinations took place in community-owned facilities that were rented for the purpose of the survey.

### Data collection procedures

Data collection in DEGS1 was based on: (1) a semi-quantitative self-administered food frequency questionnaire (FFQ); (2) automated assessment of currently used medications (AmEDa); (3) collection of urine and blood samples; (4) standardized measurements and physical performance tests (M/T); (5) a standardized physician-administered computer-assisted personal interview (CAPI); (6) a standardized self-filled questionnaire (SFQ).

The FFQ questionnaire was mailed to study respondents along with the confirmation letter. It included instructions and examples for completion. Participants were asked to take the completed questionnaire to the survey appointment.

AmEDa as well as standardized measurements and tests were conducted by study technicians. Unique product identifiers (Pharmazentralnummer, PZN) on original drug containers brought to the survey site were scanned and automatically coded according to the latest version of the WHO Anatomical Therapeutic Chemical (ATC) Classification System [14]. Information on medication source (doctor’s prescription, over-the–counter, family medicine cabinet), indication, dose, frequency and duration of use, and intake within the past 24 hours was documented. Missing information due to incomplete matching (about 27% of scanned drugs) or missing original containers (about 1.5% of participants) was completed by telephone follow-up contacts and hand searching.

Body weight and body height were measured in underwear with shoes removed. Waist and hip circumferences were measured according to a standardized protocol. Three consecutive automated blood pressure measurements were taken on the right arm three minutes apart with participants sitting and having been at rest for five minutes.

Spot urine samples were collected and dipstick tested on site. Venous blood samples were collected using Vacutainer EDTA and gel tubes. The time of blood draw and number of hours since last meal were recorded. Participants who came in fasting were served a breakfast or snack thereafter. Blood was centrifuged and separated. Serum specimens as well as urine specimens were aliquoted and stored at −40°C within one hour. EDTA whole blood tubes were shaken and kept in the original collection tubes at 4°C. A 100 μl whole blood aliquot intended for folate analysis was hemolyzed under light protection and stored at −40°C. A full blood count was performed on site. Additional whole blood, serum and urine specimens were transported by car at −40°C at the end of each survey week for further analysis and storage at the central epidemiology laboratory unit at the Robert Koch Institute, Berlin. Clinical chemistry and allergic sensitization analyses were performed within 6–8 weeks. Specific analyses and confirmatory tests relating to the diagnosis of infectious diseases were commissioned to reference laboratories. Extra serum, urine and whole blood aliquots were stored at −40°C.

Applying the CAPI, study physicians obtained a detailed medical history including family history. The interview was administered in German language, hence participants had to be sufficiently proficient in speaking and understanding German. Self-administered questionnaires filled in at the survey site were available in different languages (German, Turkish, Serbo-Croatian, Russian, English). Foreign language versions were offered to participants with German as a foreign language who were able to communicate in German, but had limited capacities for reading and writing in German. Different versions of the questionnaire were used for the population of working age (up to 64 years of age) and for persons 65 years and above. A short version of the self-administered questionnaire restricted to core questions was offered to those who lacked the strength or time to answer the full-length questionnaire.

Completing the entire survey program required an average of three hours. GNHIES98 participants who had moved outside their PSU and were unable to come to the survey site were asked to complete a computer-assisted telephone interview (CATI) covering CAPI questions and to answer a self-administered postal questionnaire.

### Objectives

DEGS1 aims to analyze health status, health risks and resources, functional capacity levels, and disability in the German adult population. Prevalence estimates will be obtained and time trends will be analyzed in comparison with data from the GNHIES98. Changes in health status and risk factors over time at the individual level will be studied among DEGS1 participants who already took part in the GNHIES98. Major study objectives include:

(1) To estimate the prevalence of diseases and risk factors with high public health impact and to identify differences according to socio-demographic characteristics, region of residence as well as changes over time;

(2) To analyze data on medication use and health care services utilization with regard to aspects of treatment effectiveness and quality of care;

(3) To investigate the association between mental health, physical health, and health-related behavior;

(4) To study patterns and determinants of co- and multimorbidity in the population 65 years and older in association with functional capacity levels, disability and health-related quality of life;

(5) To analyze allergic sensitization patterns and associations with manifest allergic disease in the population;

(6) To assess nutritional health risks and states of nutritional deficiency based on semiquantitative assessment of dietary habits and serum concentrations of various micronutrients (25-hydroxyvitamin D, vitamin B 12, iron, and ferritin in serum; serum and erythrocyte folate; urinary sodium excretion);

(7) To estimate iodine intake at the population level based on urinary iodine excretion;

(8) To examine individual changes in health status and cardiometabolic risk factors (e. g. body mass index; glycosylated hemoglobin A1c, serum lipids) over time.

### Constructs and instruments

As summarized in Table 2, constructs and instruments applied in DEGS1 were kept compatible to those used in the GNHIES98 as far as possible. The CAPI covered a total of 32 health conditions including hepatitis (A, B, C, E), prevalent chronic diseases and cardiovascular events (stroke, myocardial infarction). Participants were asked whether a particular health condition was ever diagnosed by a physician, and if so, at what age or in what year the diagnosis was first made, and whether the condition required any current medical treatment or medication use. Persons with a lifetime history of a particular disease were asked, whether the disease was still present during the 12 months preceding the interview. Disease specific information was also obtained.

**Table 2:** Constructs and instruments in DEGS1

**Construct**	**Instrument/Method**	**Data collection mode**	**Information obtained for all study participants**	**Comparable information available from GNHIES98**
**Self-reported morbidity**				
Presence of any long-standing illness	MEHM [35]	SFQ	yes	no
Medical history including family history	Self-developed closed-ended questions	CAPI	yes	yes
				(except for family history)
Lifetime history of selected childhood diseases and vaccinations	Self-developed closed-ended questions; taking copies of immunization records	CAPI	yes	yes
		Information obtained from immunization records		
**Medication use**				
Current medication use (past 7 days)	On-site medication review and automated barcoding [8]	AmEDa	yes	yes
**Objective health measures**				
Body weight and height	Portable electronic scales(SECA, Germany), stadiometer (Holtain, UK); accuracy of measurement: 0.1 kg and 0.1 cm	M/T	yes	yes
Waist and hip circumference	Flexible, non-stretchable measurement tape (Siber Hegner, Switzerland); accuracy of measurement: 0.5 cm	M/T	yes	yes
Blood pressure and heart rate	Automated blood pressure device (Datascope Accutor Plus, The Netherlands)	M/T	yes	yes
				(instrument change)
Thyroid volume	Ultrasound (USD 200 Sirius, ROI Medical Systems, Germany)	M/T	yes	no
Biochemical measures	Complete blood count^**a**^, clinical chemistry, immunology and allergic sensitization immunoassays^**b**^, spot urine dipstick^**c**^, special serum and urine analyses^**d**^	M/T	yes	yes
				(change in methods for some measures)
**Symptoms and complaints**				
Physical symptoms (past 7 days)	SCL-90-R, 12-item somatization scale [36]	SFQ	yes	no
Joint pain (past 12 months; past 24 hours)	Closed-ended questions adapted from RADAI [37]	SFQ	yes	no
Pain intensity and interference with activities of daily life (past 4 weeks)	SF-36 [38,39]	SFQ	yes	yes (SF-36 version 1.0) [40]
Sleep duration and sleep disorders	Closed-ended questions adapted from PSQI [41]	SFQ	yes	no
Urinary incontinence	Self-developed single closed-ended question	SFQ	yes	no
**Mental Health**				
Depressive symptoms	PHQ-9 [42,43]	SFQ	yes	no
(past 2 weeks)				
Chronic stress	12-item TICS screening-Scale [44,45]	SFQ	participants 18–64 years	no
(past 3 months)				
Vitality	Vitality subscale, SF-36 version 2.0 [38,39]	SFQ	yes	yes (SF-36 version 1.0) [40]
Mental distress	MHI-5 [46]	SFQ	yes	yes (SF-36 version 1.0) [40]
Psychological distress attributed to critical life events	Closed-ended questions adapted from DEAS 2002 [47]	SFQ	yes	no
Assaults and aggressive behavior (past 12 months and life time history since age 16 years)	Self-developed closed-ended questions	SFQ	participants 18–64 years	no
**Subjective health**				
Self-rated health status	MEHM [35]	SFQ	yes	SF-36 version 1.0 [40]
	SF-36 version 2.0 [38,39]			
Health-related quality of life	SF-36 version 2.0 [38,39]	SFQ	yes	SF-36 version 1.0 [40]
**Gender-specific health issues**				
Benign prostate disease	Self-developed closed-ended single question	SFQ	men of all age goups	no
Women’s health (reproductive history, hysterectomy, contraceptive use, postmenopausal hormone therapy)	Self-developed closed-ended questions	SFQ	women of all age groups	no
**Injuries**				
Injuries requiring medical treatment (past 12 months)	Self-developed closed-ended single question	SFQ	yes	yes
History of fractures (since age 50 years, selected locations, associated degree of trauma)	Closed-ended questions adapted from European Osteoporosis Study [48]	SFQ	participants 65 years and older	no
**Falls**				
History of falls (past 12 months, past 4 weeks,); fear of falls	Closed-ended questions adapted from Prevention of Falls Network Europe consensus [49]	SFQ	participants 65 years and older	no
**Functional capacities**				
Visual and hearing impairment	Closed-ended questions adapted from DEAS 2002 and ECHIM Project [47,50]	SFQ	yes	no
Aerobic fitness	Submaximal endurance bicycle ergometer testing based on heart rate and lactic acid measurements [51,52]	M/T	participants 18–64 years	no
Isometric hand grip strength	Smedley, S dynamometer	M/T	participants 65 years and older	no
	(Stoelting Co.Illinois, USA) [53,54]			
Mobility	Timed “Up & Go” [55]	M/T	participants 65 years and older	no
Balance and lower extremity strength	Balance test sequence, five-chair rise [56-58]	M/T	participants 65 years and older	no
Cognitive functioning	DSST [59,60]	M/T	participants 65 years and older	no
**Disability**				
Officially recognized disability	Self-developed closed-ended single question	SFQ	yes	yes
Long-standing illness interfering with activities of daily life	MEHM [35,61]	SFQ	yes	no
Physical functional limitations interfering with activities in daily life	Physical functioning scale, SF-36 version 2.0 [38,39]	SFQ	yes	SF-36 version 1.0 [40]
**Health-related behavior**				
Dietary habits (past 4 weeks)	Self-developed, validated semi-quantitative food frequency questionnaire (FFQ) [16]	SFQ	yes	FFQ (past 12 months; less food items; no portion sizes)
Smoking history, passive smoking, intention to quit, nicotine dependence	Self-developed closed-ended questions; FTND [62]	SFQ	yes (nicotine dependence restricted to participants 18–64 years)	yes
				(except for FTND and intention to quit)
Alcohol consumption (frequency and amount)	Closed-ended self-developed questions; AUDIT-C, BASIC [63]	SFQ	yes	yes (except AUDIT, BASIC)
Physical activity and sports activities	Self-developed closed-ended questions	SFQ	yes	partly
Media use (hours per day on average)	Self-developed closed-ended questions	SFQ	yes	no
**Living and social conditions**				
Self-perceived social support	3-item Oslo scale [64]	SFQ	yes	no
Level and type of support with activities of daily life	Self-developed closed-ended questions	SFQ	participants 65 years and older	no
Caregiving (duration; caregiver burden)	Self-developed closed-ended questions	SFQ	yes	no
Housing conditions (size and type of housing; indoor mold; noise and traffic exposure)	Self-developed closed-ended questions	SFQ	yes	yes
Working conditions (hours of work per week; shift or night work)	Self-developed closed-ended questions	SFQ	participants 18–64 years	yes
**Socio-demographic context variables**				
Age, year of birth, marital status, household size, current family structure, residence in former GDR as of 1988	Self-developed closed-ended questions	SFQ	yes	yes
Immigration background	Closed-ended questions adapted from [65]	SFQ	yes	yes
Education, net household income, professional status and employment history, retirement	Closed-ended questions adapted from [17,18]	SFQ	yes	yes
**Health care services utilization**				
Type of health insurance	Self-developed closed-ended questions	SFQ	yes	yes
Nursing care enrollment or pending application	Self-developed closed-ended questions	SFQ	participants 65 years and older	no
Last time seen a doctor; number of doctor visits, emergency care visits, hospital nights, sick days, sickness leave days (past 12 months)	Self-developed closed-ended questions	SFQ	yes	yes
Participation in cancer screening programs including specific programs (ever, last time participated)	Self-developed closed-ended questions	CAPI	yes	participation in general only (no information on utilization of specific programs)
Preventive health check-up program (past 2 years)	Self-developed closed-ended questions	SFQ	participants 18–64 years	yes
Preventive (past 12 months) and rehabilitation services (past 3 years)	Self-developed closed-ended questions	SFQ	yes	yes
Disease-specific preventive services, procedures and diagnostic tests	Self-developed closed-ended questions	CAPI	yes	no

^**a**^Cell-DYN^®^Emerald^TM^(Abbott, Germany).

^**b**^Architect^®^ ci8200 (Abbott, Germany), IC1000 (ThermoFischer, prev. Phadia, Germany).

^**c**^Combur 9 Test^®^(Roche) and Micral-Test^®^(Roche).

^**d**^Antibody titer serum concentrations (hepatitis E, measles, mumps, rubella, herpes simplex type 1 and type 2, salmonella, borrelia); PCR-based diagnostic tests in serum (hepatitis B and C) and urine (chlamydia, gonorrhoea); urinary iodine (modified Sandell-Kolthoff method) [66] and salt excretion.

*Abbreviations: AmEDa* automated medication assessment, *CAPI* computer-assisted personal interview, *M/T* measurements and tests, *SFQ* self-administered questionnaire, *AUDIT-C/BASIC* Alcohol Use Disorders Identification Test – alcohol consumption questions, Brief Alcohol Screening Instrument for Medical Care, *DEAS* German Aging Survey, *DSST* Digit Symbol Substitution Test, *ECHIM* European Community Health Indicator Monitoring, *FTND* Fagerström Test for Nicotine Dependence, *MEHM* Minimum European Health Module, *MHI-5* 5-item Mental Health Inventory, *PHQ-9* 9-item depression module of the Patient Health Questionnaire, *PSQI* Pittsburgh sleep quality index, *RADAI* Rheumatoid Arthritis Disease Activity Index, *SCL-90-R* Symptom Checklist-90-Revised, *TICS* Trier Inventory for the Assessment of Chronic Stress.

In addition to self-reported morbidity, detailed information on current medication surveyed by AmEDa, and various objective health measures will contribute to analyze health status and health risks in the adult population. Infectious diseases and immunization status will be evaluated based on self-reported childhood infections and vaccinations, immunization records, and laboratory test results. These include serum concentrations of antibody titers against several viruses and bacteria as well as nucleic acid amplification testing for confirmatory diagnosis of hepatitis B and C in serum and diagnosis of gonorrhea and chlamydia infections in urine. Medication use and objective health measures will help to validate self-reported chronic health conditions, to identify previously unknown disease and hidden health risks, and to analyze patterns of co- and multimorbidity as well as aspects of treatment effectiveness and quality of care. A list of chronic health conditions covered in DEGS1 and information available from interview and examination is given in Table 3.

**Table 3:** **Chronic health conditions and information available from health interview**^**a**^**and examination**^**b**^**in DEGS1**

**Health condition**	**Information**
**Allergic disease**	
Allergic contact eczema; allergic rhinitis; food allergy; atopic eczema; insect venom allergy; urticarial; other	Medical history and family history; current treatment; lifetime history of allergy testing and specific immunotherapy
	Inhalation allergy screening tests, total serum IgE, allergen-specific IgE antibody tests against 52 specific allergens (food, pollen, contact, insect, inner room allergens)
**Cardiovascular disease**	
Angina pectoris or other coronary heart disease	Medical history, current treatment; angina episodes and related hospitalizations, emergency room treatments, doctor visits during past 12 months; lifetime history of specific tests and surgical procedures
Myocardial infarction (MI)	Medical history and family history; current treatment; number of events; time of first and last MI; lifetime history of specific tests and surgical procedures
Heart failure	Medical history; current treatment; acute exacerbations and related hospitalizations, emergency room treatments, doctor visits within past 12 months
Peripheral artery disease	Medical history; current treatment
Stroke	Medical history and family history; current treatment; number of events, time of first and last event
**Cardiometabolic conditions**	
Diabetes mellitus	Medical history including type of diabetes and family history; diagnosis during pregnancy; current treatment; treatment following diagnosis; presence of long-term diabetes complications; history of acute hyper- or hypoglycemia and related hospitalizations, emergency room treatments, doctor visits within past 12 months; self-monitoring of blood glucose; time of last follow-up care (foot inspection, HbA1c assessment, eye background exam)
	HbA1c; serum concentrations of glucose, insulin, C-peptide, glycosuria, ketonuria, microalbuminuria
Hypertension	Medical history and family history; current treatment; self-monitoring; last time of blood pressure measurement by health professional; last self-measurement; history of hypertensive emergency and related hospitalizations, emergency room treatments, doctor visits during past 12 months
	Blood pressure measurements
Dyslipidemia	Medical history; current treatment; serum total cholesterol, LDL- and HDL- cholesterol, triglycerides
Gout and hyperuricemia	Medical history; current treatment; serum uric acid
**Gastrointestinal disease**	
Gastric or duodenal ulcer; chronic inflammatory bowel disease	Medical history; current treatment
Liver, gallbladder, pancreatic disease	Medical history of hepatitis including type of hepatitis (A, B, C, E) and liver cirrhosis; current treatment
	Hepatitis immunoassay and PCR-based confirmatory test results for hepatitis B and C
	Serum transaminases (ALT, AST), cholestatic liver enzymes (AP, GGT), lipase; bilirubinuria, urobilinogenuria
**Malignant disease**	
Any type of cancer	Medical history including type of malignancy; current treatment and cancer follow-up
**Mental health disorders**^**c**^	
Anxiety disorder; depression; burnout; eating disorder	Medical history, current treatment
**Musculoskeletal disease**	
Osteoarthritis	Medical history and history of joint replacement; current treatment, specialist care; joint pain; lower back pain
Rheumatoid arthritis	Medical history; current treatment, specialist care; joint pain
	cyclic citrullinated peptide (CCP) antibody test^**d**^
Osteoporosis	Medical history; type of current treatment and specialist care; lower back pain, history of fractures and falls; fear of falls; lifetime history of bone densitometry
	Serum 25-hydroxyvitamin-D, calcium, phosphate, intact parathyroid hormone
**Neurologic disease**	
Epilepsy; Migraine;	Medical history; current treatment
Parkinson’s disease Parkinson’s disease	
**Kidney and urogenital tract disease**	
Renal insufficiency	Medical history of renal insufficiency; current treatment
	Serum creatinine and cystatin C; microalbuminuria, proteinuria
Benign prostate disease	Lifetime history including time of diagnosis and history of prostate surgery
Gynecological conditions	Lifetime history of hysterectomy and oophorectomy; benign conditions
**Lower respiratory tract disease**	
Asthma; chronic bronchitis	Medical history including type of asthma; current treatment; lifetime history of asthma-related allergy testing and specific immunotherapy; asthma related hospitalizations, emergency room treatments, doctor visits within past 12 months
**Thyroid disease**	
Hyper-, hypothyroidism; other thyroid disease	Medical history including type of thyroid disorder, thyroid surgery, radioiodine treatment; current treatment
	Thyroid volume; serum thyroid hormones and TSH, thyroid antibodies (TPO, TGA)

^**a**^Information from computer-assisted personal interviews (CAPI) except for history of benign prostate disease, gynecological conditions, history of falls and fractures, and fear of falls (self-administered questionnaires).

^**b**^Information from standardized measurements and laboratory tests.

^**c**^Information collected in DEGS1 by CAPI, not related to additional information collected in the DEGS1 mental health study module (DEGS1-MH).

^**d**^CCP antibody testing was limited to the first year of data collection.

*Abbreviations: ALT* alanine aminotransferase, *AP* alkaline phospatase, *AST* aspartate aminotransferase, *GGT* gamma-glutamyl transferase, *TGA* thyreoglobulin antibody, *TPO* thyroid peroxidase antibody, *TSH* thyroid stimulating hormone.

Information on other facets of health, such as somatic symptoms and complaints, various aspects of mental health, and subjective health was collected via self-administered questionnaires based on standardized instruments (Table 2). Self-administered questionnaires also served to collect detailed information on reproductive history among women, a history of benign prostate disease among men, injuries, and among persons 65 years and older, a history of fractures and falls as well as fear of falls.

Functional capacities were assessed by standardized questionnaires (vision and hearing impairment) and tests. Aerobic endurance fitness tests using submaximal endurance bicycle ergometry were restricted to the study population 18–64 years. Exclusion criteria were defined using the translated version of the Physical Activity Readiness Questionnaire (PARQ) [15]. Among persons 65 years of age and older, functional capacities relevant to daily life activities were assessed (Table 2). Isometric handgrip strength in both hands was measured while participants were standing upright using the Smedley S dynamometer. A simple test sequence was used to assess balance (Romberg test, semi-tandem, tandem, one-leg stand), lower leg muscle strength and coordination (five-chair rise test), and mobility (Timed “Up & Go”). Cognitive functioning was assessed using the digit symbol substitution test (DSST) from the authorized German version of the Wechsler Adult Intelligence Scale, 3rd revision (WAIS-III).

Health risks and resources covered in DEGS1 include potentially modifiable behavioral risk factors (e.g. dietary habits, tobacco use, and risky alcohol consumption), environmental factors (e.g. living and working conditions, social support, lay caregiving), socio-demographic context variables, and health care services utilization. The FFQ was self-developed and designed to estimate the frequency and amounts of 53 food groups consumed during the past 4 weeks. It was validated against two non-consecutive 24 hour recalls and showed an overall reasonable validity [16]. Compared to the FFQ applied in the GNHIES98, the DEGS1 FFQ has more food items and includes estimation of portion sizes, which permits a more quantitative estimation of food intake. The instruments also cover a different time frame. Alcohol consumption and tobacco use were assessed by self-developed questions already applied in the GNHIES98. Validated German versions of established instruments were additionally applied in DEGS1 to identify persons with risky drinking behavior or nicotine dependence. Socio-demographic variables were assessed according to standards recommended by the German Epidemiology and Preventive Medicine Associations [17]. Education questions meet the International Standard Classification of Education (ISCED) [18].

### DEGS1 Mental Health Study Module

A separate mental health study module (DEGS1-MH) was implemented for in depth assessment of mental health disorders, help-seeking behavior and cognitive function. The data were collected using computer-assisted face-to-face interviews administered by specifically trained psychologists. DEGS1 participants were asked for their consent to be recontacted for a study center or at home appointment within four weeks after the basic DEGS1 clinic visit.

The core instrument of DEGS1–MH is a computer-assisted German version of the Munich Composite International Diagnostic Interview (DIA-X/M-CIDI) [19], a modified and translated version of the World Health Organization Composite International Diagnostic Interview (CIDI) [20]. The CIDI is a fully structured interview for the assessment of mental disorders according to the definitions and criteria of ICD-10 and DSM-IV. It consists of several sections tailored to assess mood disorders (major depression, dysthymic disorder, bipolar disorder); anxiety disorders (panic disorder, specific phobias, agoraphobia, generalized anxiety disorder, social phobia); post-traumatic stress disorder; obsessive-compulsive disorder; substance use disorders (alcohol abuse and dependence, abuse and dependence of pharmaceuticals, nicotine dependence); eating disorders; somatoform disorders (pain disorders, undifferentiated somatoform disorder); psychotic symptoms (screening without further differential diagnosis); treatment of mental disorders. The CIDI is a diagnostic instrument intended for use in clinical practice as well as in clinical and epidemiological research. It is suitable for use in population-based studies to estimate the prevalence of mental disorders, to assess severity of mental disorders and associated burden of disease, and to analyze treatment patterns, treatment barriers, and aspects of quality of care [20,21].

Cognitive function was assessed with a neuropsychological test battery that covers important cognitive domains such as episodic memory, working memory, executive function, and cognitive speed. The battery consists of established cognitive tests that were used in epidemiological studies before and were mostly derived from widely-used test batteries such as the German version of the CERAD battery [22,23].

The DEGS1-MH is carried out in close cooperation between the Robert Koch Institute and the Institute of Clinical Psychology and Psychotherapy, Center of Clinical Epidemiology and Longitudinal Studies (CELOS) at the Technische Universität Dresden. CELOS was already commissioned to carry out the Mental Health Module of the GNHIES98 [24,25]. This opens the perspective for analyses of changes in the prevalence of mental health problems as well as for longitudinal analyses based on individuals participating in both surveys.

### Assessment of non-response bias

Persons sampled and eligible for DEGS1 who declined participation were asked to provide the reason for non-participation. In addition, they were asked to fill in a short questionnaire covering essential health-related characteristics, such as self-rated health, history of long-standing chronic illness, smoking habits, educational background, and doctor visits. This will permit comparison of participants and non-participants with respect to age, sex, education, health status, health-related behavior, and health care services utilization.

### Data management and preparation for analysis

The DEGS1 data set will comprise approximately 2000 items per study participant. Data management has been carried out in parallel with data collection based on highly standardized and partly automated procedures for data processing and plausibility checking. This will assist in providing high quality and timely data. A final set of key data was available for main pre-planned analyses 6 months after the completion of fieldwork. Data from the DEGS1 survey wave will be made available to the scientific community as a public use file in 2014.

In order to assure that estimates derived from DEGS1 are representative at the national level, two sets of sample weights were computed :one set for all participants aged 18–79 years, one set for those with examination data available. Sample weights adjust for different sampling probabilities within the design strata and correct for deviations between the design-weighted net sample and German population statistics (December 31, 2010) based on an iterative raking procedure using age, sex, federal state of residence, community BIK classification category, nationality (German yes/no), and education level according to ISCED [18] using population statistics dating from 2009. For GNHIES98 participants included in DEGS1, the weighting procedure additionally includes adjustments for the re-participation rate.

### Quality assurance

The DEGS1 study operations manual contains standard operating procedures (SOPs) for all DEGS1 interview and examination components as well as internal and external quality control measures [8]. Members of the study teams underwent an initial two-week training session leading to accreditation. They were continuously supervised and reassessed at 6-month intervals. Internal quality control was carried out by senior RKI supervising staff. Measures of internal quality assurance encompassed: regular field visits to the study teams, bimonthly reviews of logbook entries for meeting requirements of daily calibration procedures and transportation of blood and urine specimens, continuous data checks for completeness and plausibility. External quality assurance was carried out by specifically experienced scientists of the Bremen Institute for Epidemiology and Prevention Research (BIPS) based on field observation and data audits.

### Feed-back to study participants

Participants received immediate feed-back on clinically relevant test results based on written summary reports prepared by study physicians. Within six to eight weeks, study participants received a standardized letter summarizing clinically relevant study results including clinical chemistry, test results for hepatitis, chlamydia and gonorrhea infections, specific allergy testing, body mass index, blood pressure, thyroid volume, and physical fitness. Study participants will also be informed about immunity status against poliomyelitis, measles, mumps, and rubella as test results become available.

### Collaborations

The DEGS1 steering committee at the Robert Koch Institute welcomes collaborations with external researchers. Collaborations may relate to input of specific know-how and professional expertise to analyses that were given a particularly high level of priority and therefore pre-planned to be completed within two years after finishing fieldwork. Additional analyses will require a research proposal, which will be reviewed by the DEGS1 steering committee and the Robert Koch Institute Research Council in order to ensure data protection and privacy regulations, public health relevance, data efficiency, and scientific quality. For further information please visit the DEGS1 study homepage at http://www.degs-studie.de.

## Discussion

As part of the recently established continuous health monitoring system in Germany, the first data collection wave of the German Health Interview and Examination Survey for Adults (DEGS1) was conducted by the Robert Koch Institute between November 2008 and December 2011. The DEGS national health survey system is primarily designed to provide nationally representative health interview and examination data for the population 18–79 years of age and to conduct analyses of time trends in population health and disability. Interview and examination data will be collected in periodically repeated survey waves at 8-year intervals [6]. By including a large number of persons who already participated in a previous national health interview and examination survey (GNHIES98), DEGS1 combines a national health survey and a survey follow-up study. In order to keep cross-sectional results of DEGS1 representative at the national level, a number of measures were taken. First, to compensate for attrition and aging of the GNHIES98 cohort, a sample of the population 18–79 years was newly drawn from local population registries using a two-stage stratified cluster sampling procedure. Secondly, results from DEGS1 cross-sectional analyses will be weighted by survey-specific weights considering sampling probabilities and non-response for newly drawn participants as well as re-participation probability for GNHIES98 participants.

In DEGS1, a wide range of objective health measures were collected based on highly standardized measurements and tests including laboratory analyses. Automated medication assessment and coding provided detailed and objective information on current medication use. It will hence be possible to verify self-reported health conditions, to identify undetected cases of disease, and to evaluate current disease or risk factor control in the population. High risk groups and health inequalities will be identified based on analyses stratified by sex, age, educational background, income, professional and employment status, and region of residence. Burden of disease will be assessed by analyzing the association of morbidity and comorbidity with functional impairment, subjective health, health-related quality of life, and disability. In order to explore preventive potential, these associations will be further analyzed with respect to personal and environmental resources, e.g. socio-demographic characteristics, health-related behavior, level of perceived social support, health care services utilization.

Results of trend analyses will be used to identify new health risks, to evaluate the effectiveness of health programs and regulations at the population level (e.g. reduction of smoking rates; improved hypertension control), and to validate absolute risk prediction models. Interpretation of time trends will need to consider survey data as well as additional information, such as trends in cause-specific mortality, disease management program enrollment, treatment patterns, ambulatory care sensitive hospital admissions, and nursing care rates. As an example, the prevalence of persons with known diabetes mellitus is likely to have increased over time due to aging of the population and increases in the prevalence of major diabetes risk factors such as obesity. However, improved awareness, treatment and survival may have contributed to increases in prevalence as well. Further insight will be gained from comparative analyses of time trends in the prevalence of persons with known and undetected diabetes.

With respect to time trend analyses, concepts, indicators, instruments and methods of data collection in DEGS1 were kept the same as in prior national health interview and examination surveys, in particular the GNHIES98, as far as possible. There were certain limitations to this. For example, there was a change in methods for measuring blood pressure and some biochemical measures. Analyses of time trends will hence require cross-calibration studies in some cases. Furthermore, several constructs and instruments were newly added in DEGS1. Health indicators have been continuously reviewed and extended in close cooperation with health policy makers and public health expert groups, in order to permit harmonization with regional population-based studies in Germany and with national health surveys from other countries. In order to improve the comparability of results between countries, great efforts have been undertaken at the EU and OECD level to harmonize indicators and instruments and to rigorously standardize the data collection process [26-29]. Areas of ongoing work on indicators particularly relate to health in older age [30].

DEGS1 response rates among newly recruited study participants are considerably lower than response rates in the GNHIES98 (42% vs. 62%). Declining response rates in population-based health surveys have consistently been reported from other European countries over the past decade [31,32]. Selection bias resulting from selective participation of healthier persons is a concern in any population-based survey [33]. Survey results may therefore underestimate the overall prevalence of chronic diseases and disability compared to results from claims data [34]. In addition, persons unable to provide written consent and those with significant language barriers were excluded from participation in DEGS1. Thus, DEGS1 is likely to underrepresent adults living in institutions. To some extent, this may also be true for persons with an immigration background, even though sample weights encompass adjustment for nationality (German yes/no). Careful non-response analyses will be carried out based on available information from official health statistics and non-response questionnaires with respect to nursing care enrollment and institutionalization, non-German nationality, self-rated health, history of long-standing chronic illness, smoking habits, educational background, and health care services utilization.

About half of the DEGS1 study population already participated in a previous national health survey (GNHIES98). This provides ten- to 12-year follow-up data on nearly 4000 individuals. It will hence be possible to analyze age- and sex-specific individual level changes in many risk factors and health status indicators available from both surveys as presented in Table 2[8,16-18,35-66]. The DEGS survey panel will continuously grow and timepoints of measurements will be added as DEGS participants who agreed to be re-contacted will be included in subsequent survey interview and examination waves. In between examination survey waves, DEGS participants will be contacted by telephone and postal questionnaires, in order to assess incident health events and changes in health status and health-related behavior. The panel will be continuously followed for vital status and cause-specific death. Participants will be asked for advance written permission for death certificate review, in order to assess cause-specific mortality. Together, this growing data base will facilitate to study trajectories of risk factors, chronic conditions, and health outcomes. In addition, extended follow-up will increase absolute numbers for estimates of disease incidence and prospective analyses using incident health events and total or cause-specific deaths as outcome measures [6].

In summary, data from DEGS1 and subsequent DEGS survey waves will make essential contributions to the surveillance of communicable as well as non-communicable diseases in Germany. Data collected in periodically repeated national health surveys of adults in Germany will be used for continuous health reporting. Findings will be relevant to health policy planning and evaluation, public health research, and health information of the lay public. Survey participants will be followed for changes in health status and health outcomes. Cross-sectional and longitudinal data will be entered in a large health data platform for health and health care services research.

## Competing interests

The authors declare that they have no competing interests.

## Authors’ contributions

BMK, PK, CSN conceptualized and supervised the study and wrote the final manuscript. AG, ML and HH oversaw the field work and quality assurance of data, assisted in conceptualizing the study and contributed to writing the manuscript. MB, SD, RD, UE, JF, UH, CH, HK, DL, GBM, HN, AR, AS, ASR, HS, MT provided specific knowledge, contributed to the conceptualization of the study and the quality assurance of data, as well as to the writing of the final manuscript. All authors read and approved the final manuscript.

## Pre-publication history

The pre-publication history for this paper can be accessed here:

http://www.biomedcentral.com/1471-2458/12/730/prepub
